# Supplementary material for: Small molecule inhibition of ATM kinase increases CRISPR-Cas9 1-bp insertion frequency
Source: Nat Commun. 2021 Aug 25;12:5111. doi: 10.1038/s41467-021-25415-8 (PMC8387472; doi:10.1038/s41467-021-25415-8)
Supplement: Supplementary file 1 — Supplementary Information [file 41467_2021_25415_MOESM1_ESM.pdf]

Supplementary Fig. 1: Schematic of the small molecule screen

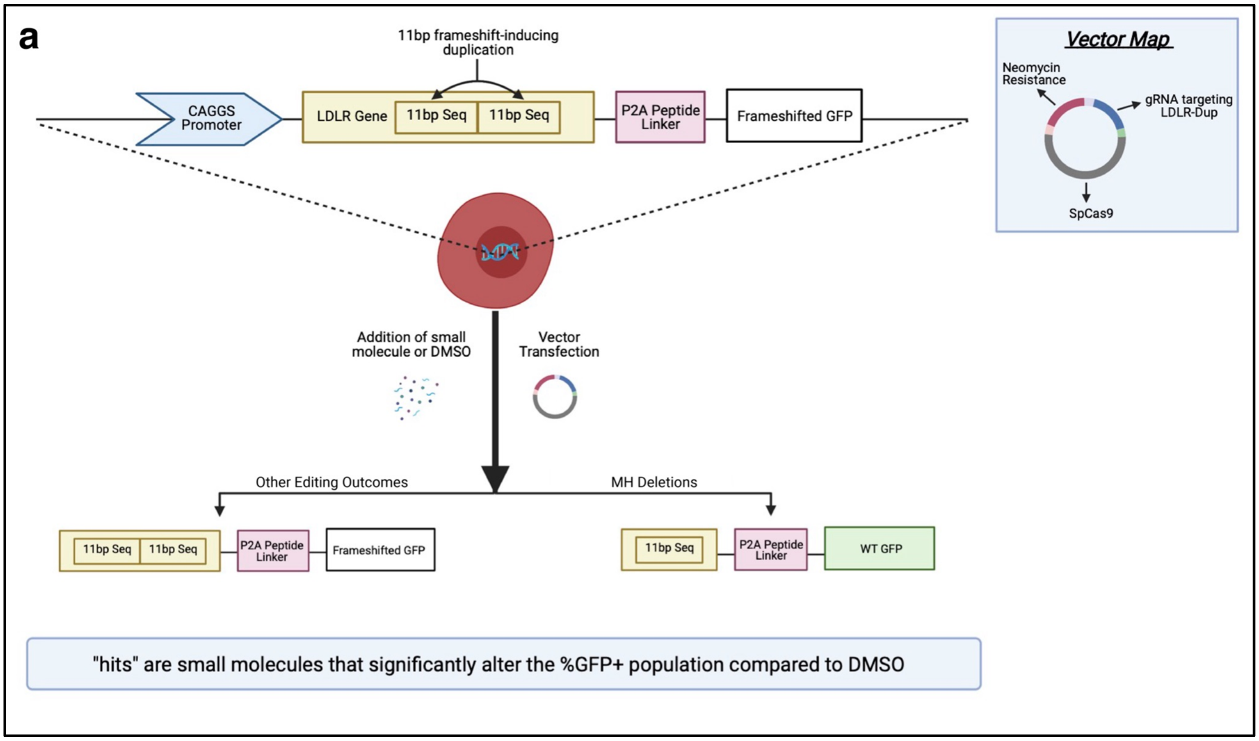

a) Schematic of the original 487 small-molecule screen shown in Fig. 1a,b. U2OS or mESC cells containing the LDLR1662-1669dupGCTGGTGA-P2A-GFP(LDLR-Dup) construct[1] were transfected with a vector containing Cas9 and an LDLRdup-targeting gRNA in the presence of a unique small molecule or DMSO, and the % of GFP positive cells post-editing were determined via FACS analysis in order to determine the relative frequency of MH-deletion events. This schematic was created on BioRender.com by co-author Sammy Barkal.

Supplementary Fig. 2: Outcome of a screen for small molecules altering CRISPR-Cas9 mutational outcomes in mESC.

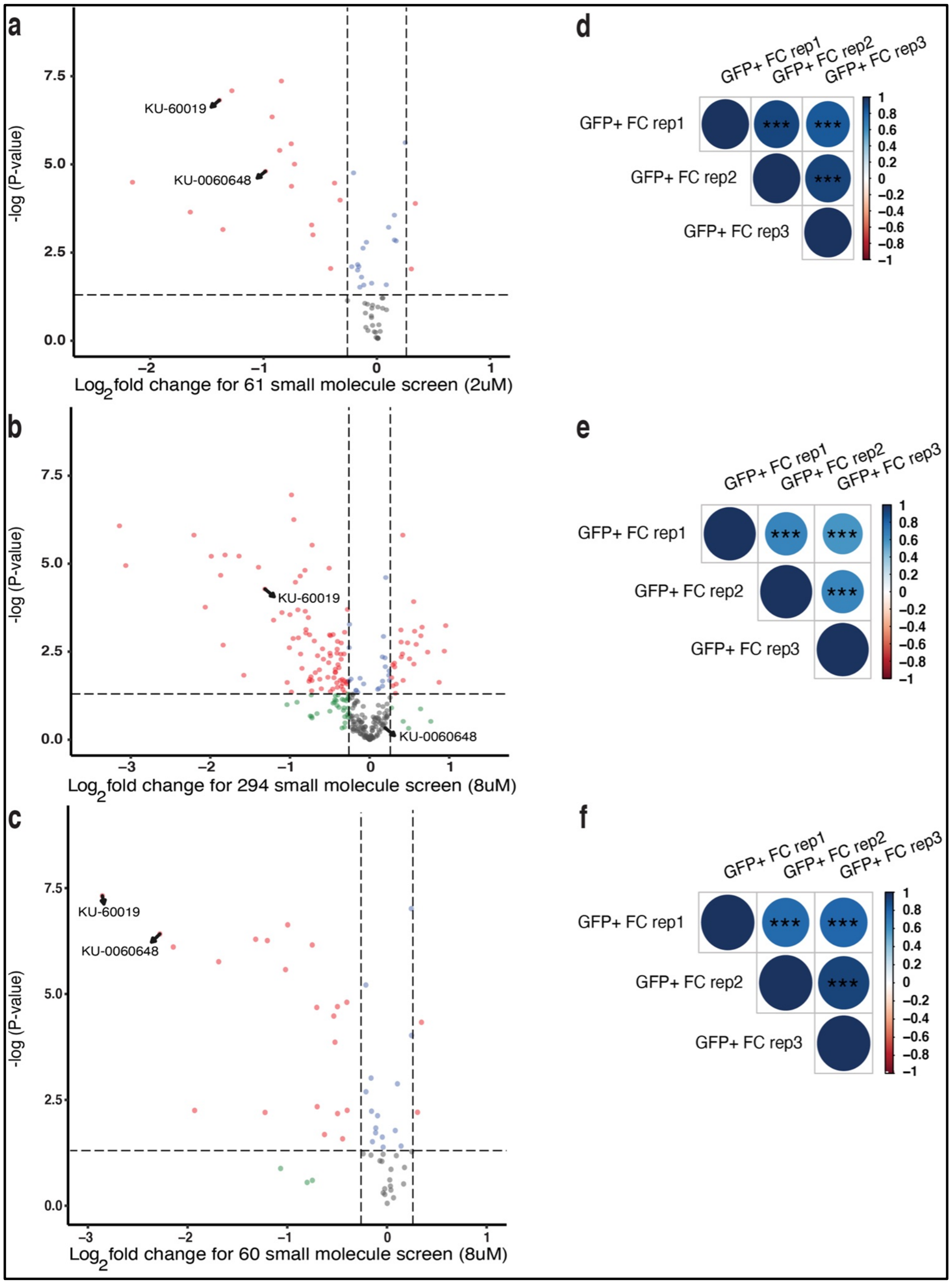

a-c) Volcano plots exhibiting the log2(FC) in GFP+ cells relative to the DMSO control for the 61 small molecule screen at 2uM (a), the 294 small molecule screen at 8uM (b), and the 60 small molecule screen at 8uM (c), respectively (N=3 biologically independent samples). P values were calculated using a two-sided welch t-test. **d-f)** Spearman correlation figures portraying the replicate consistency for screens in (a),(b), and (c) respectively. *** signifies $p<0.001$.

Supplementary Fig. 3: FACS gating strategy for 487 small-molecule screen

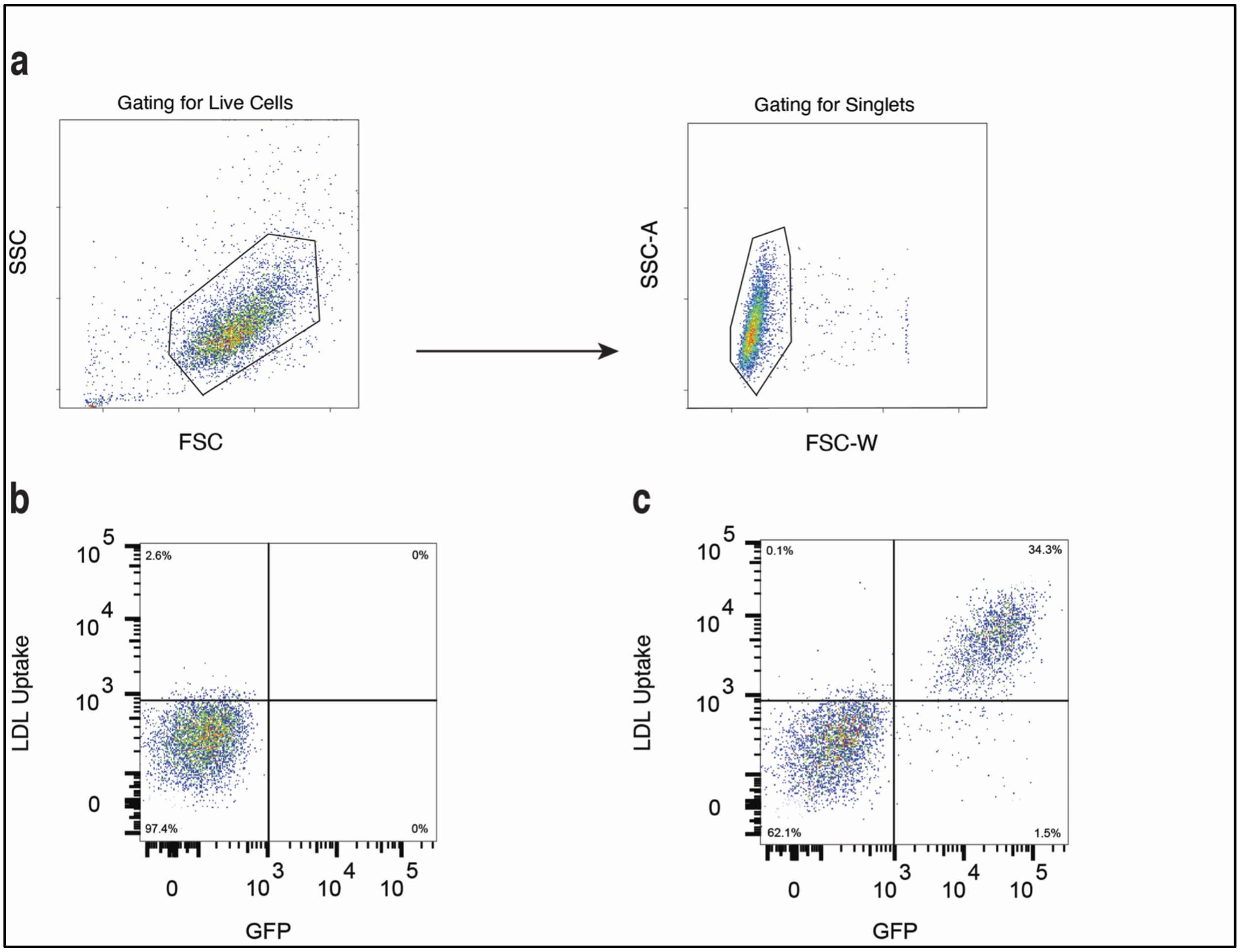

a) panel depicting the initial gating for healthy single cells. **b)** panel depicting the LDL-uptake and GFP fluorescence levels of cells containing the LDLR-Dup construct prior to the addition of Cas9 **c)** panel depicting the LDL-uptake and GFP fluorescence levels of cells containing the LDLR1662-1669dupGCTGGTGA-P2A-GFP(LDLR-Dup) construct post-Cas9 addition. Small Molecule hits were defined as those whose samples exhibited a significantly different fraction of GFP+cells (Upper right quadrant in the gating depicted).

Supplementary Fig. 4: KU-60019 and KU-0060648 do not induce significant cell death in the context of CRISPR-Cas9 editing.

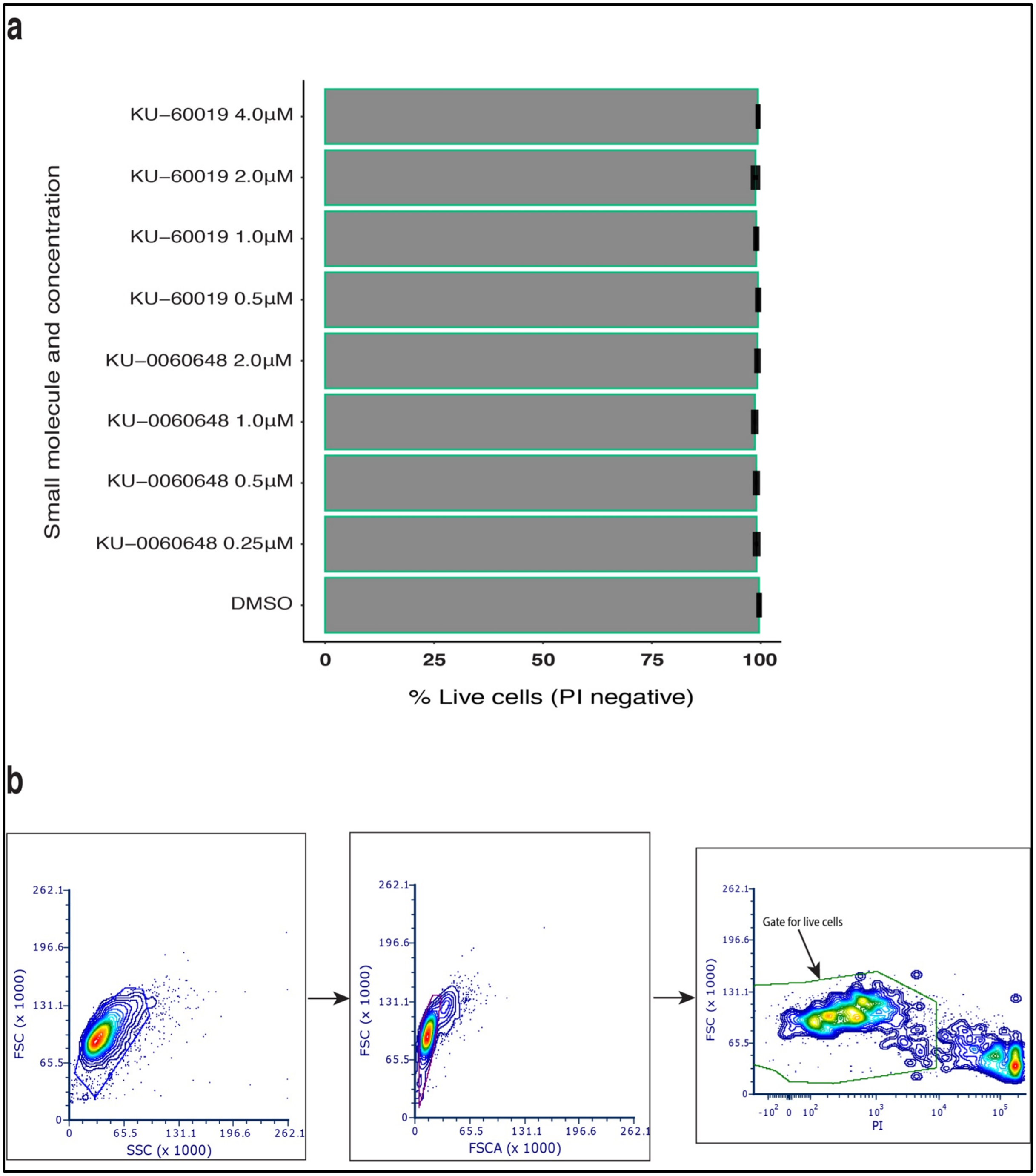

a) Percent of gated live mESC cells (PI negative) after 24hr treatment with KU-60019, KU-0060648 and DMSO. N=3 biologically independent samples, except for KU-60019-0.5µM and KU-0060648-1µM (N=2 biologically independent samples). Error bars reflect +/- the SEM. **b)** FACS gating strategy for PI staining.

Supplementary Fig. 5: KU-60019 significantly increases SpCas9-NG and KKH-SaCas9 1-bp insertion fraction at pathogenic human 1-bp deletion alleles.

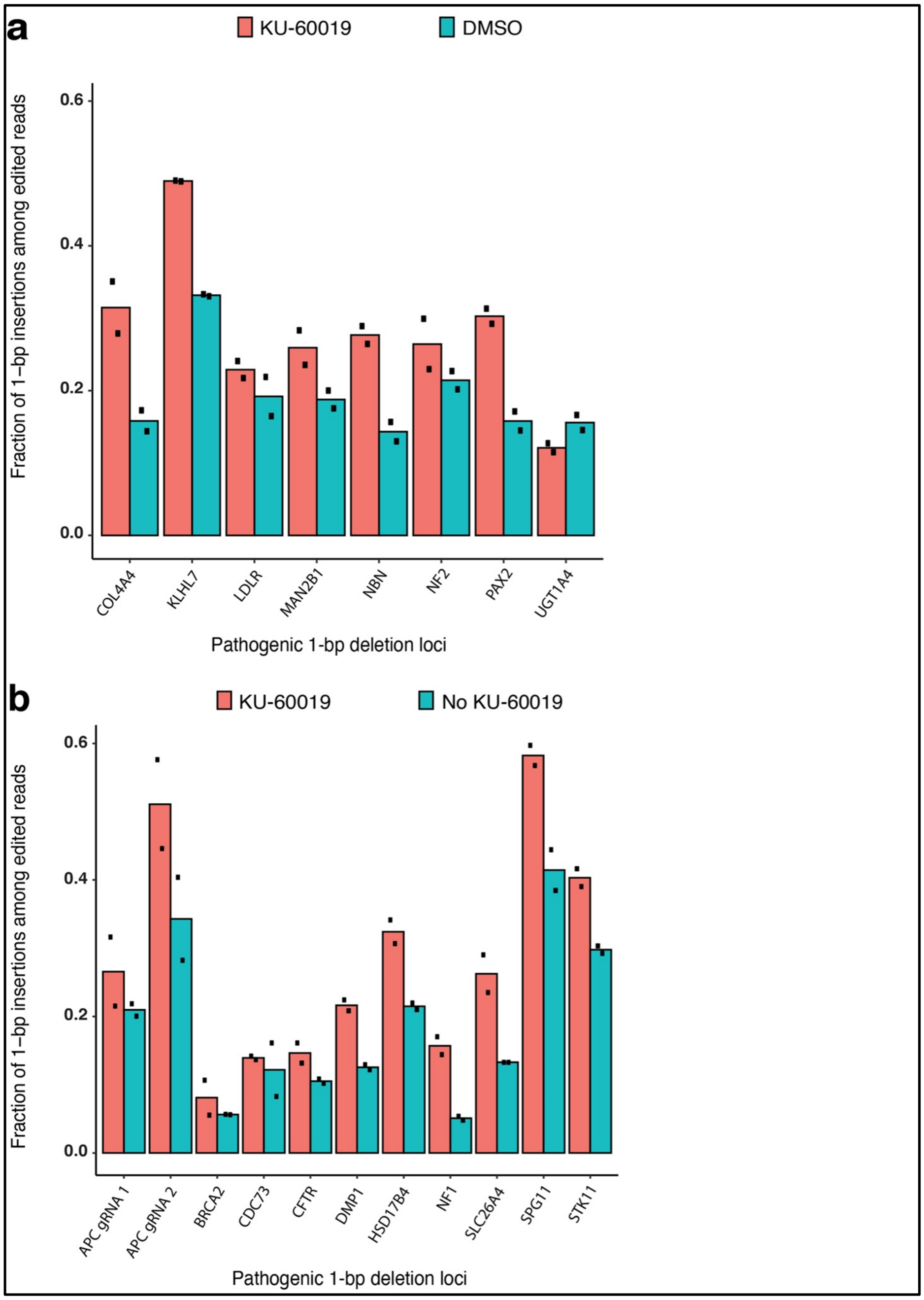

a) Fraction of 1-bp insertions among edited reads at 8 pathogenic 1-bp deletion loci included in the 48-site library and targeted by SpCas9-NG gRNAs in mESCs (N=2 biologically independent samples). **b)** Fraction of 1-bp insertions among edited reads at 11 pathogenic 1-bp deletion loci included in the 12-site library and targeted by KKH-SaCas9 gRNAs in mESCs (N=2 biologically independent samples). As a group, both SpCas9 and KKH-SaCas9 alleles have significantly more 1-bp insertion repair outcomes in the presence of KU-60019.

Supplementary Fig. 6: KU-60019 increases 1-bp insertions when delivered as a ribonucleoprotein complex.

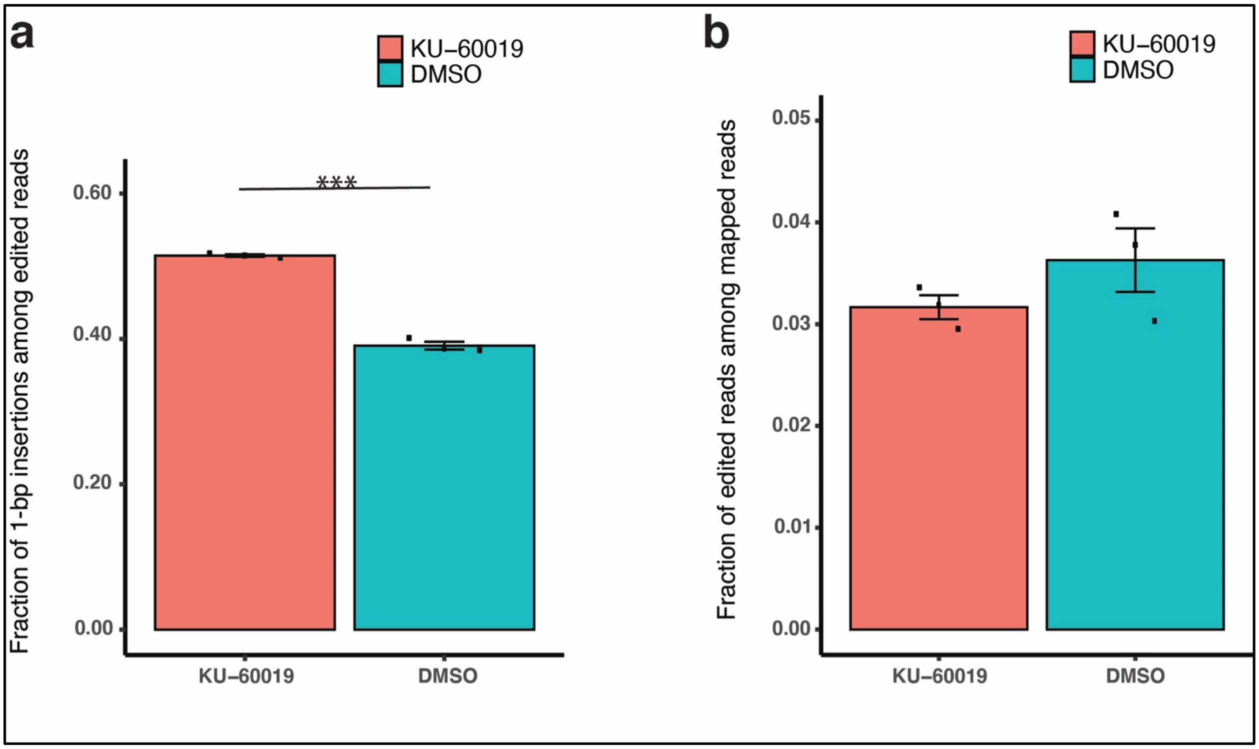

a-b) Bar charts showing (a) the fraction of 1-bp insertions out of all edited reads using SpCas9 ribonucleoprotein (RNP) targeting of mCherry, either in the presence of 2uM KU60019 or DMSO, and (b) the fraction of edited alleles in this experiment. P values were calculated using a two-sided Welch's t test (N=3 biologically independent samples). *** signifies p< 0.001. Error bars reflect +/- the SEM.

Supplementary Fig. 7: KKH-Cas9 and Sp-Cas9NG screens at native loci demonstrate KU-60019's ability to increase the frequency of 1-bp insertions at native sites with high overall editing efficiency.

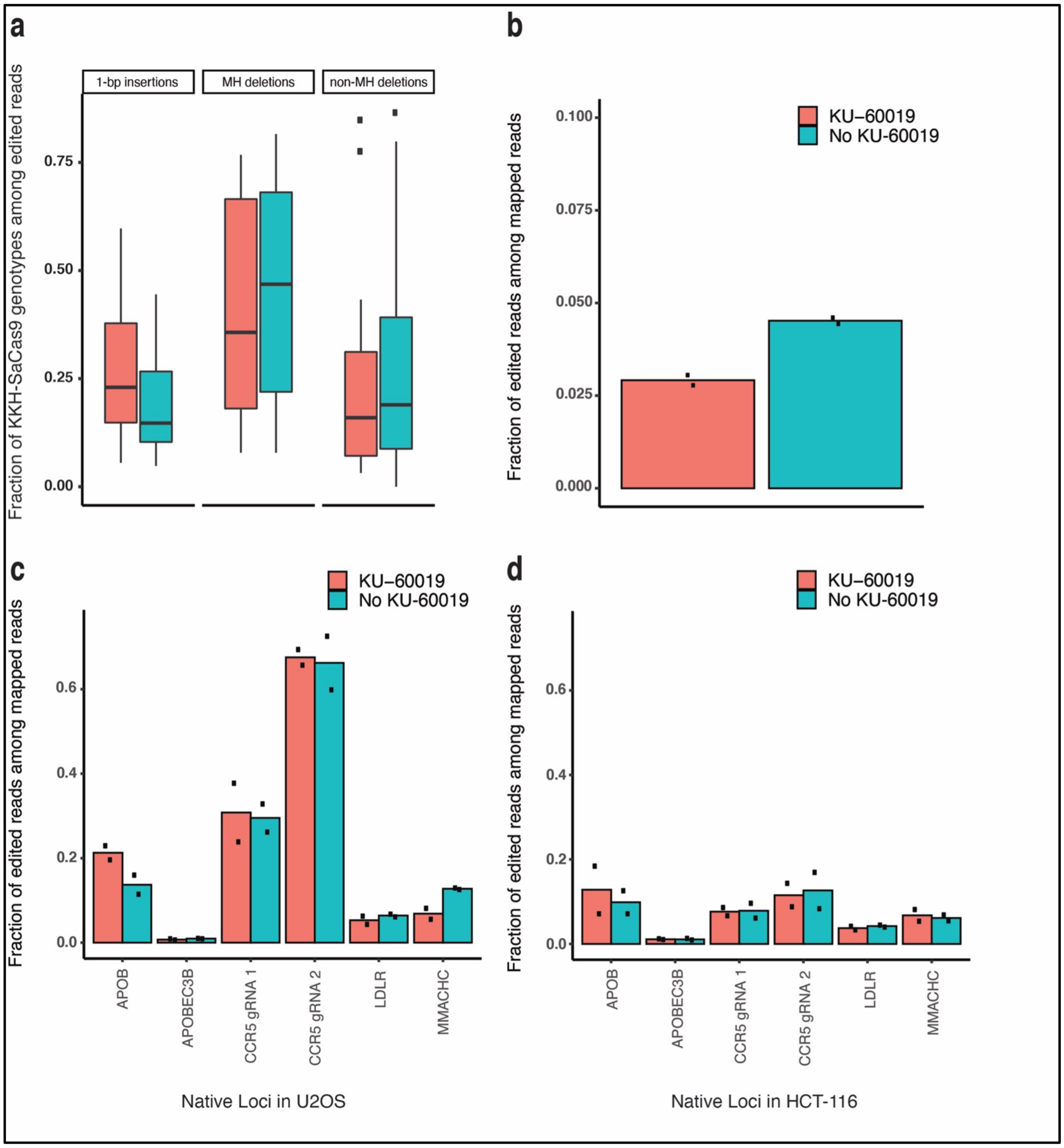

a) Boxplot exhibiting the fraction of 1-bp insertion, MH deletion, and non-MH deletion outcomes among edited reads for targets within the 12-site KKH-SaCas9 library. N=2 biologically independent samples, 11 gRNAs with sufficient data to be depicted. **b)** Bar chart exhibiting the KKH-SaCas9's overall editing efficiency for all 11-gRNA depicted in panel a **c-d)** bar charts exhibiting the overall editing efficiency of SpCas9-NG among all mapped reads at noted Precision$_{50}$ genomic loci in U2OS (c) and HCT-116 (d), N = 2 biologically independent samples. The P-value for panel a was calculated using a two-sided t-test.

Boxplot statistics for 1-bp insertions with KU-60019: minima =0.0554, lower whisker bound=0.0554, lower box bound=0.144, center=0.230, upper box bound=0.390, upper whisker bound=0.597, maxima=0.597.

Boxplot statistics for 1-bp insertions without KU-60019: minima =0.048, lower whisker bound=0.048, lower box bound=0.102, center=0.147, upper box bound=0.282, upper whisker bound=0.444, maxima=0.444.

Boxplot statistics for MH deletions with KU-60019: minima=0.079, lower whisker bound=0.079, lower box bound=0.179, center=0.357, upper box bound=0.708, upper whisker bound=0.768, maxima=0.768.

Boxplot statistics for MH deletions without KU-60019: minima =0, lower whisker bound=0, lower box bound=0.086, center=0.189, upper box bound=0.401, upper whisker bound=0.865, maxima=0.865.

Boxplot statistics for non-MH deletions with KU-60019: minima=0.032, lower whisker bound=0.032, lower box bound=0.069, center=0.160, upper box bound=0.316, upper whisker bound=0.432.

Boxplot statistics for non-MH deletions without KU-60019: minima=0.079, lower whisker bound=0.079, lower box bound=0.218, center=0.468, upper box bound=0.702, upper whisker bound=0.815.
