# Supplementary material for: Small molecule inhibition of ATM kinase increases CRISPR-Cas9 1-bp insertion frequency
Source: Nat Commun. 2021 Aug 25;12:5111. doi: 10.1038/s41467-021-25415-8 (PMC8387472; doi:10.1038/s41467-021-25415-8)
Supplement: Supplementary file 2 — Description of Additional Supplementary Files [file 41467_2021_25415_MOESM2_ESM.pdf]

Description of Additional Supplementary Files

Supplementary Data 1: LDLR-Dup construct and targeting gRNA sequences

Exact sequences of the LDLR-Dup construct and targeting gRNA.

Supplementary Data 2: Small molecule library information

Names, catalog numbers, and known functions for each small molecule within the 487-molecule SelleckChem library utilized for the small molecule screen.

Supplementary Data 3: Raw data of the 487 small molecule 2 uM library screen

Raw data of the 2uM screen for the 487-small molecule library.

Supplementary Data 4: 38 small molecule hits from the initial screen

38-small molecule hits from the initial 487-small molecule library screen that induced a significant difference in MH-deletions.

Supplementary Data 5: 48-site gRNA/target information

Target sequences and gRNA sequences for each gRNA:target site pair within the 48-site library.

Supplementary Data 6: Genotypic data for 48-site library screen

Number and fraction of observed MH deletions, non-MH deletions, 1-bp insertions, multibase insertions, and non-edited reads for 38-small molecules tested with the 48-site library screen in U2OS cells and mESC cells.

Supplementary Data 7: ClinVar/HGMD analysis for repairing pathogenic 1-bp deletions

Analysis of pathogenic 1-bp deletions from the ClinVar and HGMD databases, using inDelphi to predict the frequency with which each variant can be corrected to wild-type genotype through template-free SpCas9 editing.

Supplementary Data 8: ClinVar/HGMD analysis for installing pathogenic 1-bp insertions

Analysis of pathogenic 1-bp insertions from the ClinVar and HGMD databases, using inDelphi to predict the frequency of introducing ClinVar/HGMD pathogenic 1-bp insertions using SpCas9.

Supplementary Data 9: KKH-SaCas9 12-site library information

gRNA and target sequences within the KKH-SaCas9 12-site library.

Supplementary Data 10: Full list of primers utilized in this study

Sequences and descriptions of primers utilized within the study.

Supplementary Data 11: Human Genome Precision50 gRNA and locus information

Six Precision50 gRNAs used within this study, the sequences of the genomic PCR primers used to amplify the loci targeted, and other relevant information about the target sites.
