# Supplementary material for: Small molecule inhibition of ATM kinase increases CRISPR-Cas9 1-bp insertion frequency
Source: Nat Commun. 2021 Aug 25;12:5111. doi: 10.1038/s41467-021-25415-8 (PMC8387472; doi:10.1038/s41467-021-25415-8)
Supplement: Supplementary file 14 — Reporting Summary [file 41467_2021_25415_MOESM14_ESM.pdf]

nature research

Corresponding author(s): Richard Sherwood

Last updated by author(s): Jul 25, 2021

Reporting Summary

Nature Research wishes to improve the reproducibility of the work that we publish. This form provides structure for consistency and transparency in reporting. For further information on Nature Research policies, see our Editorial Policies and the Editorial Policy Checklist.

Statistics

For all statistical analyses, confirm that the following items are present in the figure legend, table legend, main text, or Methods section.

n/a	Confirmed	
☐	☒	The exact sample size (*n*) for each experimental group/condition, given as a discrete number and unit of measurement
☐	☒	A statement on whether measurements were taken from distinct samples or whether the same sample was measured repeatedly
☐	☒	The statistical test(s) used AND whether they are one- or two-sided<br>*Only common tests should be described solely by name; describe more complex techniques in the Methods section.*
☒	☐	A description of all covariates tested
☐	☒	A description of any assumptions or corrections, such as tests of normality and adjustment for multiple comparisons
☐	☒	A full description of the statistical parameters including central tendency (e.g. means) or other basic estimates (e.g. regression coefficient) AND variation (e.g. standard deviation) or associated estimates of uncertainty (e.g. confidence intervals)
☐	☒	For null hypothesis testing, the test statistic (e.g. *F*, *t*, *r*) with confidence intervals, effect sizes, degrees of freedom and *P* value noted<br>*Give P values as exact values whenever suitable.*
☒	☐	For Bayesian analysis, information on the choice of priors and Markov chain Monte Carlo settings
☒	☐	For hierarchical and complex designs, identification of the appropriate level for tests and full reporting of outcomes
☐	☒	Estimates of effect sizes (e.g. Cohen's *d*, Pearson's *r*), indicating how they were calculated

Our web collection on statistics for biologists contains articles on many of the points above.

Software and code

Policy information about availability of computer code

Data collection	BD FACSDiva was used to collect flow cytometry data
Data analysis	Custom python code used for data analysis is available at "https://github.com/maxwshen/indelphi-dataprocessinganalysis". Crispresso2 was used for analysis of 1bp insertions in human genome loci. FCS Express version 7 Research or FlowJo version 10.5. 0 was used for fluorescence analysis.

For manuscripts utilizing custom algorithms or software that are central to the research but not yet described in published literature, software must be made available to editors and reviewers. We strongly encourage code deposition in a community repository (e.g. GitHub). See the Nature Research guidelines for submitting code & software for further information.

Data

Policy information about availability of data

All manuscripts must include a data availability statement. This statement should provide the following information, where applicable:

- Accession codes, unique identifiers, or web links for publicly available datasets
- A list of figures that have associated raw data
- A description of any restrictions on data availability

Processed data associated to manuscript figures have been deposited at DOI 10.6084/m9.figshare.12844577, 10.6084/m9.figshare.12844625, 10.6084/m9.figshare.12844631, 10.6084/m9.figshare.14046581.
Sequencing data fastq files have been submitted to NCBI under submission Bioproject ID: PRJNA744770 and PRJNA658607

Field-specific reporting

Please select the one below that is the best fit for your research. If you are not sure, read the appropriate sections before making your selection.

☒ Life sciences ☐ Behavioural & social sciences ☐ Ecological, evolutionary & environmental sciences

For a reference copy of the document with all sections, see nature.com/documents/nr-reporting-summary-flat.pdf

Life sciences study design

All studies must disclose on these points even when the disclosure is negative.

Sample size	Sequencing depth was chosen to obtain a mininum of 1000 reads per designed oligo based on empirical measurements of the distribution of coverage over library oligos.
Data exclusions	When calculating statistics of interest on processed data, subset of data with less than 300 reads were excluded. Exclusion criteria was not pre-established.
Replication	At least two experimental replicates were carried out for each set of treatment/control samples. All experimental replicates were successful.
Randomization	Randomization was not relevant to our study. The impact of cell to cell variability is reduced to insignificant amounts by obtaining a minimun cell population diversity of about 1000 cells for per designed library oligo .
Blinding	Blinding was not relevant to our study. Treatment data was adjusted using control data

Reporting for specific materials, systems and methods

We require information from authors about some types of materials, experimental systems and methods used in many studies. Here, indicate whether each material, system or method listed is relevant to your study. If you are not sure if a list item applies to your research, read the appropriate section before selecting a response.

Materials & experimental systems

n/a	Involved in the study	
☒	☐	Antibodies
☐	☒	Eukaryotic cell lines
☒	☐	Palaeontology and archaeology
☒	☐	Animals and other organisms
☒	☐	Human research participants
☒	☐	Clinical data
☒	☐	Dual use research of concern

Methods

n/a	Involved in the study	
☒	☐	ChIP-seq
☐	☒	Flow cytometry
☒	☐	MRI-based neuroimaging

Eukaryotic cell lines

Policy information about cell lines

Cell line source(s)	All human cell lines were obtained from ATCC, U2OS, HCT116. Mouse embryonic cell lines were originally obtained as a gift from Hynek Wichterle at Columbia University, and were derived from Michael Kyba's lab originally.
Authentication	Human cell lines were not authenticated since they were obtained from ATCC. Mouse embryonic cell lines were authenticated by RNA-seq profiling.
Mycoplasma contamination	Cells were tested every month for mycoplasma contamination and were always negative.
Commonly misidentified lines (See ICLAC register)	No commonly misidentified cell lines were used.

Flow Cytometry

Plots

Confirm that:

☒ The axis labels state the marker and fluorochrome used (e.g. CD4-FITC).

☒ The axis scales are clearly visible. Include numbers along axes only for bottom left plot of group (a 'group' is an analysis of identical markers).

☒ All plots are contour plots with outliers or pseudocolor plots.

☒ A numerical value for number of cells or percentage (with statistics) is provided.

Methodology

Sample preparation	For small molecule screens, LDLR-dup mESC were treated with fluorescent LDL (LDL-Dylight 550) and gated as previously described in Shen, Arbab et al. Nature,2018.<br>mESC with 48 gRNA member library treated with small molecules at different doses were incubated with Propidium Iodide (PI) for 30 minutes to measure live/dead cells. Then, cells were trypsinized, filtered through 0.22 uM filter, and run through the flow cytometer
Instrument	BD Biosciences FACS symphony
Software	BD Bioscieces FACSDiva
Cell population abundance	No cell sorting was carried out.
Gating strategy	Cells were first gated via FSC-A and SSC-A to defined main population. Cells were next gated via SSC-A and SSC-W to define single cells.<br>For small molecule screen with LDL-dup mESC, gating strategy is shown in Shen, Arbab et al. Nature,2018.<br>For mESC with 48 gRNA member library treated with small molecules at different doses, Live and dead population were obtained by setting gate 4 between negative control (PI, no treatment) and positive control (PI + triton 0.25% treated cells).

☒ Tick this box to confirm that a figure exemplifying the gating strategy is provided in the Supplementary Information.
